# Supplementary material for: New Pharmacological Strategies against Pancreatic Adenocarcinoma: The Multifunctional Thiosemicarbazone FA4
Source: Molecules. 2022 Mar 4;27(5):1682. doi: 10.3390/molecules27051682 (PMC8911630; doi:10.3390/molecules27051682)
Supplement: Supplementary file 1 [file molecules-27-01682-s001.zip › molecules-1555477-supplementary.pdf]

Supplementary Materials

New Pharmacological Strategies against Pancreatic Adenocarcinoma: The Multifunctional Thiosemicarbazone FA4

Dario P. Anobile [1,2], Mauro Niso [3], Adrian Puerta [2,4], Stephanie M. Fraga Rodrigues [2], Francesca S. Abatematteo [3], Amir Avan [5,6], Carmen Abate [3,*,†], Chiara Riganti [2,7,*,†] and Elisa Giovannetti [2,8,*,†]

[1] Department of Oncology, University of Torino, Via Santena 5/bis, 10026 Torino, Italy; dario.anobile@edu.unito.it
[2] Department of Medical Oncology, Amsterdam UMC, VU University, Cancer Center Amsterdam, De Boelelaan 1117, 1081HV Amsterdam, The Netherlands; apuertaa@ull.edu.es (A.P.); s.m.fragarodrigues@amsterdamumc.nl (S.M.F.R.)
[3] Dipartimento di Farmacia-Scienze del Farmaco, Università degli Studi di Bari ALDO MORO, Via Orabona 4, 70125 Bari, Italy; mauro.niso@uniba.it (M.N.); francesca.abatematteo@uniba.it (F.S.A.)
[4] BioLab, Instituto Universitario de Bio-Orgánica "Antonio González" (IUBO-AG), Universidad de La Laguna, c/Astrofísico Francisco Sánchez 2, 38206 La Laguna, Spain
[5] Basic Medical Sciences Institute, Mashhad University of Medical Sciences, Mashhad 91886-17871, Iran; avana@mums.ac.ir
[6] Basic Medical Sciences Institute, Mashhad University of Medical Sciences and Metabolic Syndrome Research Center, Mashhad University of Medical Science, Mashhad 91886-17871, Iran
[7] Interdepartmental Research Center of Molecular Biotechnology, University of Torino, 10126 Torino, Italy
[8] Fondazione Pisana per la Scienza, Via Ferruccio Giovannini 13, 56017 San Giuliano Terme, Italy
* Correspondence: carmen.abate@uniba.it (C.A.); chiara.riganti@unito.it (C.R.); e.giovannetti@amsterdamumc.nl (E.G.)
† These authors contributed equally to this work.

Supplementary Figure S1

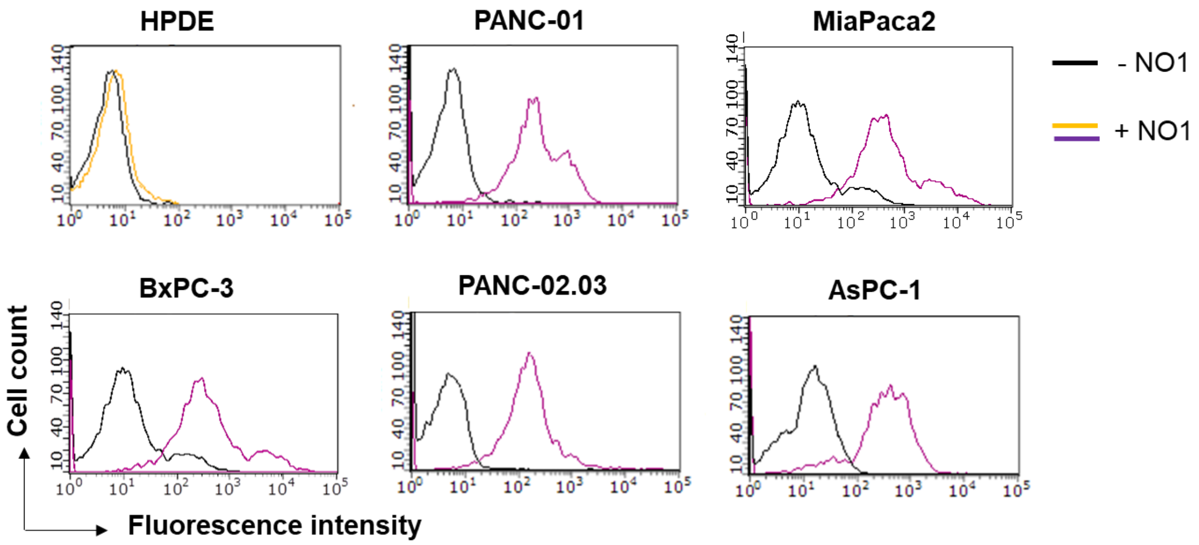

Supplementary Figure S1. Amount of plasma-membrane associated σ2-receptor on pancreatic cancer cells and non-transformed pancreatic epithelial cells. The amount of σ2-receptors present on the plasma-membrane of non-transformed HPDE cells and pancreatic ductal adonocarcioma PANC-1, MiaPaCa2, BxPC-3, PANC-02.03, AsPC-1 cells, incubated in the absence (-) or presence (+) of 100 nM NO1, a σ2 receptor fluorescent ligand, was measured by flow cytometry, in duplicates. The figures report representative istograms of three independent experiments.

Supplementary Figure S2

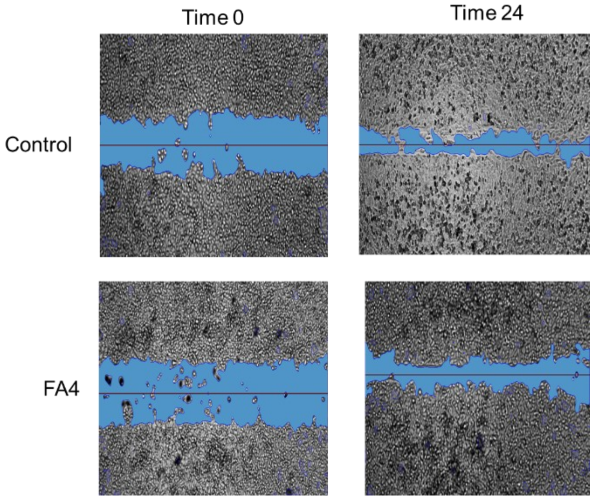

Supplementary Figure S2. Results of the migration assay performed in PDAC-1 cells. After the scratch, PDAC-2 cells were incubated in fresh medium (Control) or with **FA4** 10 μM. Representative pictures from three indepenendet experiments were taken at different time points (0 and 24 h).
